# Comparison of non-invasive strategies to drive fluid resuscitation in sepsis or septic shock: a meta-analysis of RCTs

**DOI:** 10.1007/s11739-025-04179-9

**Published:** 2025-11-05

**Authors:** Mara Graziani, Laura Gasperini, Claudia Gasperini, Giorgio Maraziti, Gennaro De Pascale, Cecilia Becattini

**Affiliations:** 1 Internal, Vascular and Emergency Medicine - Stroke Unit, University of Perugia, Perugia, Italy; 2 Department of Emergency Science, Anesthesiology, and Resuscitation, Fondazione Policlinico Universitario A. Gemelli IRCCS, Rome, Italy

**Keywords:** Capillary refill time, Echocardiography, Fluid resuscitation, Lactate clearance, Passive leg-raising, Sepsis, Septic shock, Stroke volume, Ultrasound, Usual care

## Abstract

Early fluid resuscitation reduces mortality in patients with sepsis or septic shock, but excessive fluid administration may prolong hospitalization and increase complications. Several non-invasive strategies have been proposed to guide fluid resuscitation, yet their comparative efficacy remains uncertain. We systematically searched PubMed and EMBASE through June 2025 to identify randomized controlled trials evaluating non-invasive strategies to guide fluid resuscitation in adult patients with sepsis or septic shock. Pairwise and network meta-analyses were conducted to assess short-term mortality. Length of stay (LOS) in intensive care unit (ICU) was also analyzed. 20 RCTs (2.435 patients) were included. In pairwise meta-analyses, lactate clearance-guided resuscitation was associated with reduced short-term mortality compared to ScvO₂ (RR 0.81, 95% CI 0.65–1.00; *I*^2^ = 0%). No significant difference in mortality was observed between capillary refill time versus lactate clearance (RR 0.82; 95% CI 0.65–1.04; *I*^2^ = 0%), passive leg-raising plus stroke volume versus usual care (RR 0.91, 95% CI 0.67–1.23; *I*^2^ = 14%), echocardiography versus usual care (RR 0.72, 95% CI 0.32–1.61; *I*^2^ = 70%), and inferior vena cava collapsibility versus usual care (RR 0.75, 95% CI 0.52–1.09; *I*^2^ = 19%). In network meta-analysis, none of the assessed strategies to guide fluid resuscitation reduced mortality compared to usual care. Heterogeneity was moderate. Non-invasive dynamic parameters—lactate clearance, along with capillary refill time and inferior vena cava collapsibility assessment—are promising, bedside-available tools for guiding fluid resuscitation in patients with sepsis or septic shock. Their broader use in clinical practice may be warranted, pending confirmation from high-quality studies.

**Supplementary Information:**

The online version contains supplementary material available at https://doi.org/10.1007/s11739-025-04179-9.

## Background

Sepsis accounts for 2–6% of all hospital admissions and is associated with in-hospital mortality of up to 15% [1]. Mortality can increase to 56% when sepsis progresses to septic shock with hypotension and hypoperfusion [2–4]. Early intervention is critical to limit progression to septic shock and to decrease mortality in patients with sepsis.

Based on health system organization and on patient features, patients with sepsis and septic shock can be managed in intensive care units (ICUs) or in acute care units capable of providing adequate treatment and monitoring. With this in mind, it is crucial to identify management strategies with favorable balance between accuracy and ease of use.

Prompt fluid resuscitation is essential to increase cardiac preload and mean circulatory filling pressure [5, 6].

Fluid resuscitation, together with antimicrobial therapy, limits sepsis progression to shock and reduces mortality by approximately 16% [7]. However, there's evidence indicating a risk for potential overfilling, leading to complications, such as pleural effusion, prolonged hospitalization, and increased morbidity and mortality (OR 1.92, CI 95%, 1.16–3.22) in the case of excessive fluid resuscitation [7].

Several targeted approaches have been proposed to drive fluid resuscitation. For almost twenty years, international guidelines recommended the use of central venous pressure (CVP) to guide fluid management. However, CVP's ability to predict fluid responsiveness is as low as 56%, indicating equal likelihood of fluid responsiveness regardless of CVP level [8]. Alternative dynamic parameters include variations in pulse pressure, stroke volume, and/or of systolic pressure, as well as plethysmography variability index. All these parameters necessitate advanced monitoring equipment and invasive procedures, which limit their application outside the ICU or in resource—limited settings and increase the risk of hospital-acquired infections and peri-procedural complications [9].

Recently, non-invasive dynamic strategies, easily obtainable at the bedside, have been proposed for fluid resuscitation guidance in sepsis or septic shock. These are serum lactate levels, capillary refill time, ultrasound assessments, and others [10–13]. Nonetheless, the limited evidence from randomized clinical trials (RCTs) highlights uncertainty regarding the optimal strategy.

This critical review and network meta-analysis of RCTs aimed to assess the role of non-invasive dynamic parameters to drive fluid resuscitation in patients with sepsis or septic shock.

## Methods

A critical review was first conducted to describe the complete scenario of the available RCTs on the role of non-invasive dynamic parameters to drive fluid resuscitation in patients with sepsis or septic shock. A meta-analysis and a network meta-analysis were then conducted to provide quantitative comparisons among individual non-invasive dynamic parameters to drive fluid resuscitation in this setting results.

### Data sources and searches

The study protocol for the meta-analysis was registered in PROSPERO (Prospero ID: CRD42024539351) and was developed according to the Preferred Reporting Items for Systematic Reviews and Meta-Analyses (PRISMA) Statement guidelines [14]. We systematically searched PubMed, EMBASE, and clinicalTrials.gov for studies published from 2000 to June 2025. Full search strategies are reported in the Supplementary Material. No restriction was used for language, study design, or country.

### Study selection

RCTs were considered potentially eligible if they met the following predetermined criteria: i) included adult patients with sepsis or septic shock; ii) compared different strategies to drive fluid resuscitation; iii) reported on death occurring during hospitalization or within 30 days. For duplicate publications, the most complete was considered. Three reviewers (M.G., L.G., and C.G.) independently performed study selection, with disagreements solved through discussion and the opinion of a fourth reviewer (C.B.). Kappa statistics was used to assess the inter-reviewer agreement on study selection [15].

### Study outcomes

The primary study outcome was short-term death, defined as death occurring in-hospital or within 30 days since hospitalization. Secondary study outcome was length of stay (LOS) in ICU.

### Data extraction and quality assessment

For each study, three authors (M.G., L.G., and C.G) independently extracted the following: general data (study design and year of publication), characteristics of the study population (mean age, gender, and setting of care), strategies to drive fluid resuscitation and study outcomes data (number of participants who experienced an event for dichotomous outcomes, and means or medians and measures of variability for continuous outcomes).

Study quality was assessed by two reviewers (L.G. and C.G.) using the Cochrane collaboration’s tool to assess risk of bias in randomized trials (ROB-II) [16]. We rated studies as being at high risk of bias if i) more than one domain was judged at ‘high risk of bias’ or ii) one domain was judged at ‘high’ and at least two at ‘probably high’ risk of bias. Conversely, studies were rated as low risk of bias only if all domains were judged as either probably low or low risk of bias. We resolved disagreements in study data extraction and risk of bias assessment by consensus or by discussion with an additional reviewer (M.G.).

### Statistical analysis

Estimates of pooled effect sizes were obtained by the Mantel–Haenszel method and reported as pooled risk ratios (RRs) with 95% confidence intervals (CIs) for short-term mortality and as mean differences (MDs) for ICU length of stay.

For binary outcome data with zero events in either group, a continuity correction of 0.5 was added to each cell for each effect measure. Sub-group analyses were conducted using the test for subgroup differences (chi-square test) under both fixed and random effects models.

Forest plots were created for each outcome. Results were reported according to the fixed effects model in the absence of significant heterogeneity and random effects model in the presence of significant heterogeneity. Heterogeneity was quantified using the I-squared statistics. I-squared of 50% or higher was used to define significant heterogeneity [17]. Publication bias was assessed visually by inspection of funnel plots.

Network meta-analysis was fitted to a frequentist framework using common and random effect network meta-analysis models with the statistical package *netmeta* in R statistical software version 4.0.2 (R Project for Statistical Computing) [18]. The size of the nodes in the map was proportional to the number of patients who received the intervention in the network, and the width of the edges was proportional to the number of trials that compared the two treatments.

We estimated the ranking probabilities of the different strategies based on their P scores. The P score is measured on a scale from 0 (worst) to 1 (best), with a higher score indicating better overall performance of the competing treatment. The local consistency assumption was not assessed in this network meta-analysis because there was no closed loop in our networks. However, we applied *Q* statistics to test for global inconsistency using a design-by-treatment interaction random effects model.

We assessed network heterogeneity across all treatment contrasts using τ^2^ and *I*^2^ statistics.

The certainty of the evidence was assessed independently and in duplicate using the GRADE approach for NMAs. Certainty of evidence was rated using the GRADE approach adapted to network meta-analysis through the CINeMA (Confidence in Network Meta-Analysis) framework [19].

Sensitivity analyses were conducted to assess the effects of different strategies to drive fluid resuscitation in short-term mortality on study quality.

Statistical analyses, forest plots, and funnel plots were produced using Review Manager release 5.4 (The Cochrane Collaboration, Oxford, England) and STATA/SE 12 (StataCorp LP, College Station, TX, USA).

## Results

Overall, 20.489 papers were retrieved from literature search, with 780 deemed eligible for inclusion after title and abstract review. After a full-text review, 20 RCTs (2.435 patients) comparing different strategies to guide fluid therapy were finally identified for inclusion in the meta-analyses [20–39]. The inter-reviewer agreement regarding study selection was good (kappa statistics: 0.84). Flow diagram for study selection is reported in Supplementary Material Figure S1.

### Description of studies

The main features of included studies are reported in Table 1. All were open-label studies and one study used 2:1 randomization [20]; four were conducted at emergency departments [23, 30, 36, 38], the remaining were all conducted at ICUs. Fourteen studies included patients with a septic shock, two studies patients with sepsis and the remaining four studies included either patients with sepsis or septic shock. The number of included patients varied across the studies between 42 and 424. Seven studies were rated as having a low risk of bias (Supplementary material Table S1).

**Table 1:** Baseline characteristics of the RCTs studies included in the meta-analysis

Author, years	Setting	Overall population	Male (%)	Age (mean)	Follow-up days	Septic shock/sepsis	Intervention group (patient num.)	Control group (patient num)	Death rate %
Douglas IS, 2020 [20]	ICU	124	60	61	30 days	Sepsis	PLR + SV (83)	Usual care (41) ^*^	27.7
Kuan WS,2016 [21]	ED	122	71	65	28 days	Septic Shock/sepsis	PLR + SV (61)	Usual care (61) +	25.8
*Li G, 2019 *[22]	*n.a*	*74*	*n.a*	*n.a*	*In-hospital*	*Septic shock*	*PLR* + *SV (37)*	*Usual care (37) *^*§*^	*23.3*
Richard JC, 2019 [23]	ICU	60	43	64	28 days	Septic shock	PLR + SV (30)	Usual care (30) °	64.3
Chen C,2015 [24]	ICU	82	50	59	In-hospital	Septic shock	PLR + SV (41)	Usual care (41) ^#^	29.3
Jones AE, 2010 [25]	ICU	300	54	61	In-hospital	Septic shock	LC (150)	ScvO_2_ (150)	20.0
*Lyu X, 2015 *[26]	*ICU*	*100*	*n.a*	*n.a*	*28 days*	*Septic shock*	*LC (50)*	*ScvO*_*2*_* (50)*	*19.3*
*Wang T, 2014 *[27]	*ICU*	*57*	*n.a*	*n.a*	*28 days*	*Septic shock*	*LC (17)*	*ScvO*_*2*_* (26)*	*42.0*
Puskarich MA, 2012 [28]	ED	353	54	59	In-hospital	Septic Shock/sepsis	LC (93)	ScvO_2_ (110)	19.7
*Tian H, 2012 *[29]	*ICU*	*62*	*n.a*	*n.a*	*28 days*	*Septic shock*	*LC (43)*	*ScvO*_*2*_* (19)*	*18.7*
*Yu B, 2013 *[30]	*ICU*	*50*	*n.a*	*n.a*	*28 days*	*Septic shock*	*LC (25)*	*ScvO*_*2*_* (25)*	*24.0*
Chen H, 2021 [31]	ICU	82	66	72	In-hospital	Septic shock/sepsis	LC (42)	Usual care (40) **	52.4
Hernàndez G, 2019 [32]	ICU	424	48	60	28 days	Septic shock	CRT (212)	LC (212)	39.1
Castro R, 2020 [33]	ICU	42	43	58	28 days	Septic shock	CRT (21)	LC (21)	21.4
Musikatavorn K, 2021 [34]	ED	202	57	64	30 days	Septic shock	IVC (101)	Usual care (101) ^##^	19.3
Ghosh S, 2024 [35]	ICU	166	57	56	28 days	Septic shock	IVC (83)	Usual care (83) ^§^	19.9
Sricharoenchai T, 2024 [36]	ED	138	61	70	28 days	Septic shock	IVC (69)	Usual care (69^)§^	15.2
Li L, 2021 [37]	ICU	97	54	56	28 days	Sepsis	Echo (49) ^^^	Usual care (48) ^§§^	58.0
Lanspa MJ, 2018 [38]	ICU	30	50	66	28 days	Septic shock	Echo (15) ^^^^	Usual care (15) °°	26.6
Alhabashy WS, 2021 [39]	Surgical ICU	87	54	43	30 days	Septic shock/sepsis	Echo (42) ^$$^	Usual care (45) ^$^	24.1

*ICU* Intensive care unit; *ED* Emergency department,*CRT* capillary refill time, *PLR* passive leg-raising; *SV* stroke volume;*LC* lactate clearance; *IVC* Inferior vena cava, *ScvO2* central venous oxygen saturation, *echo* Echocardiography, *n.a* not available

Studies in *italic* are those only available as abstract

^*^Fluid resuscitation driven by clinical signs, such as mean arterial pressure (MAP), low urine output, and any other clinical indication

+ Fluid resuscitation driven by MAP

^§^Not available

°Fluid resuscitation driven by MAP and CVP

^#^Fluid resuscitation driven by CVP, MAP, pulse pressure variation (PPV)

^**^Fluid resuscitation driven by the central venous pressure (CVP)

^##^Fluid resuscitation driven by physicians’ discretion

^§§^Fluid resuscitation driven by CVP

°°Fluid resuscitation driven by CVP, MAP, lactate; ScvO2 was checked to start dobutamine

^$^ Fluid resuscitation driven by CVP, MAP; ScvO2 was checked to transfuse red cells and to start dobutamine

^Echo: Sub-costal four chamber for inferior vena collapsibility; Parasternal long- and short-axis views for LV ejection fraction; Apical view for the right ventricular/Left ventricular telediastolic area; the bilateral lung ultrasound protocol: Each of the 10 covered regions was assessed for detailed pleural sliding and morphology

^^Echo: Focused Transthoracic Echocardiography to assess the inferior vena cava (IVC) collapsibility and myocardial function

^$$^ Echo: IVC Collapsibility and IVC distensibility index > 18% indicates fluid responsiveness. Passive leg-raising test plus SV

The duration of follow-up was 28 days in twelve studies, 30 days in three studies, and the overall duration of hospitalization in five studies. Mortality varied across studies from 19.3% to 58.0%. The parameters to drive fluid resuscitation in the intervention group were passive leg-raising plus stroke volume in five studies, lactate clearance in seven, capillary refill time (CRT) in two, different combinations of ultrasound assessments in three, and inferior vena cava (IVC) collapsibility in three. The comparator was a usual care in 12 studies, ScvO2 in six, and lactate clearance in two. The definition of usual care in the individual studies entailed different combinations of different parameters, the most common being mean arterial pressure (MAP), low urine output, CVP, or pulse pressure variation (PPV).

A difference in short-term mortality between intervention and controls emerged in two studies only available in abstract version, and in one study conducted in a surgical ICU comparing fluid resuscitation driven by ultrasonography with usual care. No difference in short-term mortality emerged in the remaining studies between the assessed strategies.

### Strategies to guide fluid resuscitation and associated short-term mortality

No evidence of publication bias was observed at funnel plot inspection for these analyses (Supplementary Material Figure S2).

#### Lactate clearance vs ScvO2

**Fig. 1:**
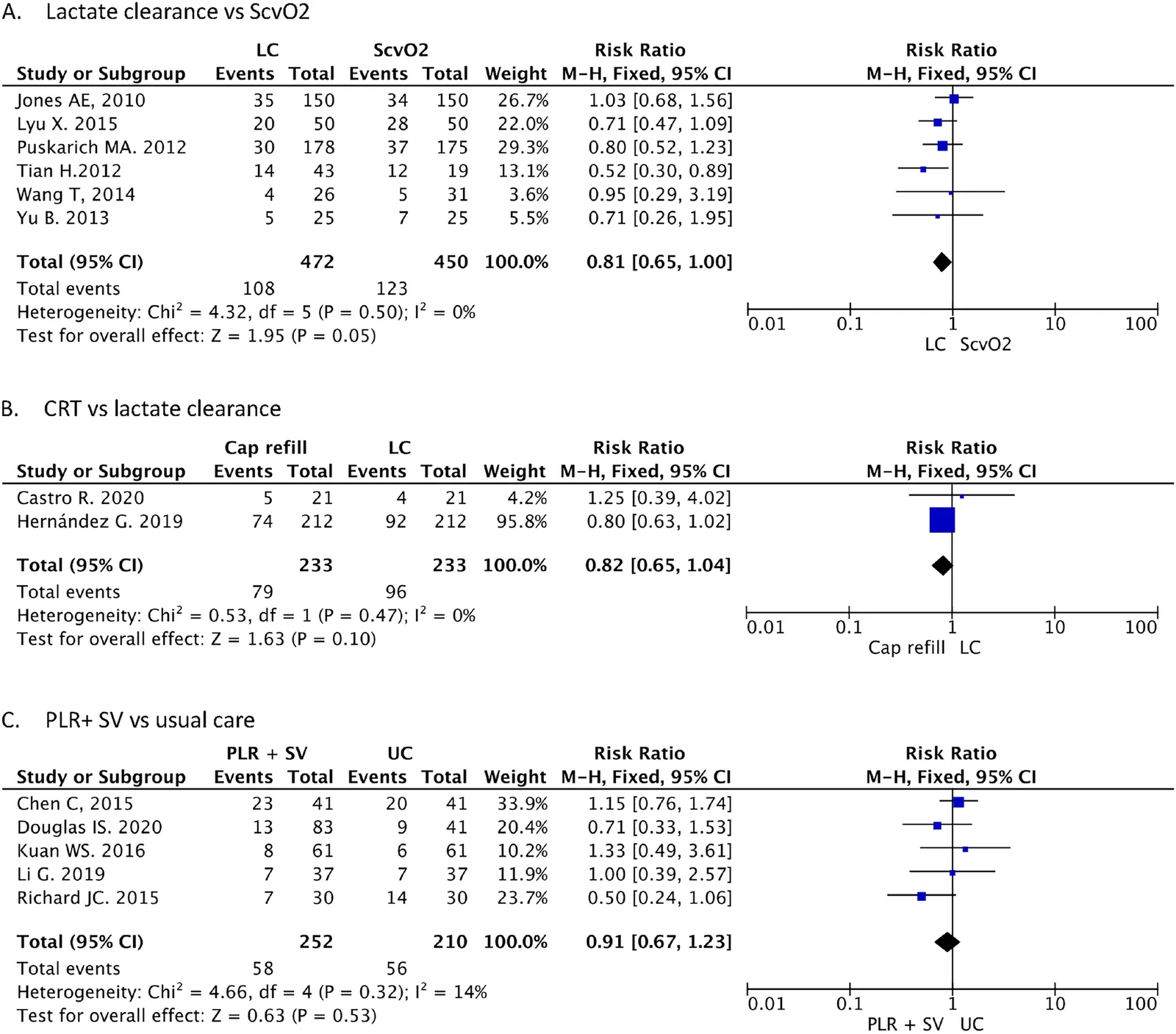

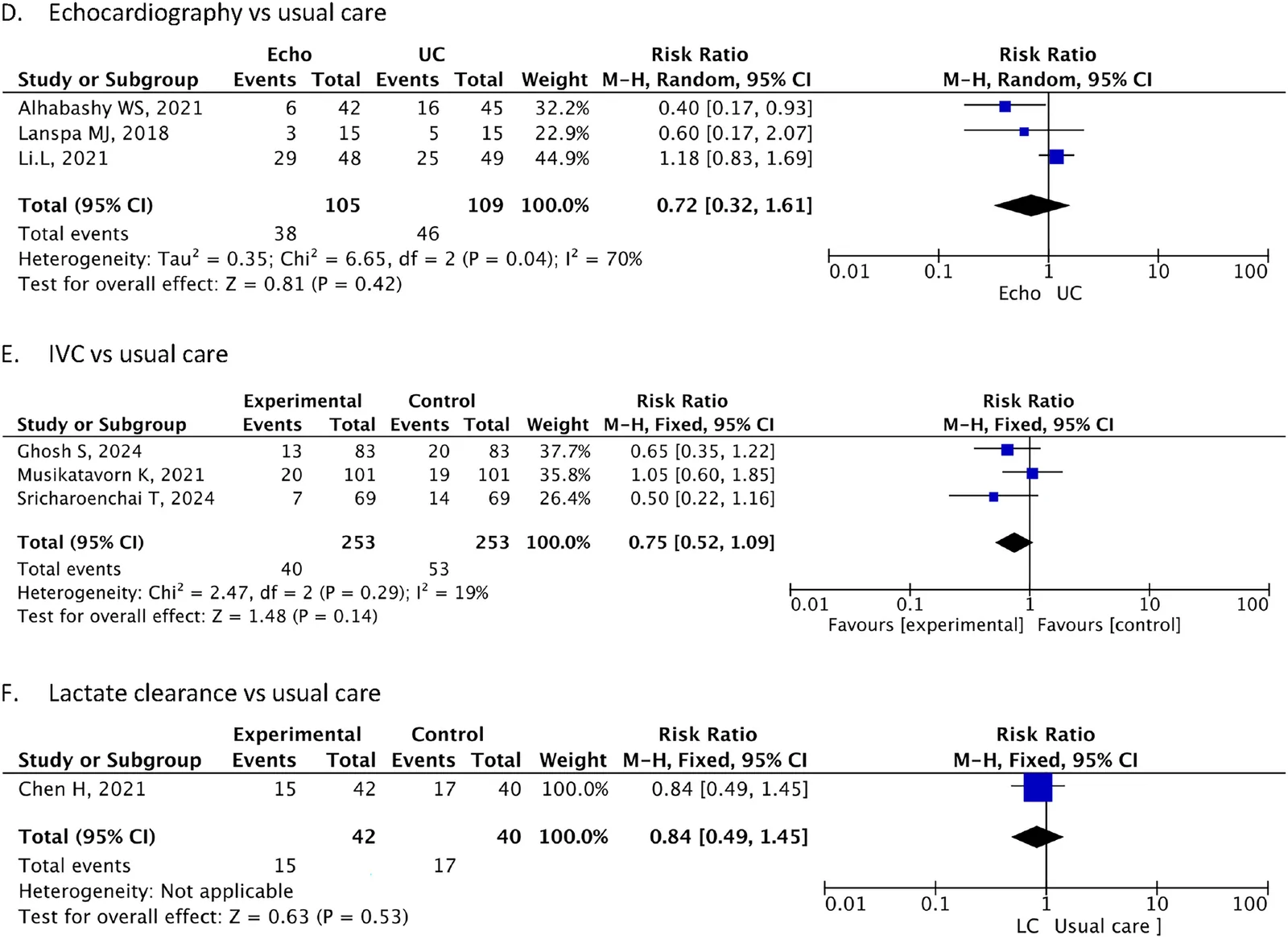
Short-term mortality in patients randomized to diffeent strategies to drive fluid resuscitation

Six studies compared lactate clearance with ScvO2 to drive fluid resuscitation (922 patients). Short-term mortality occurred in 22.9% of 472 patients randomized to lactate clearance and in 27.3% of 450 patients randomized to ScvO2, respectively. Lactate clearance-driven fluid resuscitation was associated with a reduction in short-term mortality (RR 0.81; 95% CI 0.65–1.00, *I*^2^ 0%) (Fig. 1, panel A, and Table 2). These results were not confirmed after exclusion of four studies only available in abstract version in English or Mandarin (2 studies, 503 patients; 26.7% of 243 patients randomized to lactate clearance and 27.3% of 260 patients randomized to ScvO2, respectively; RR 0.99; 95% CI 0.67–1.48, *I*^2^ 0%).

**Table 2:** Risk of short-term death by strategy for guiding fluid resuscitation

Compare strategies	Number of studies/number of patients	Short-term mortality		RR (95% CI)	I-squared
		intervention group n/N (%)	Control group n/N (%)		
LC vs ScvO2	6/922	108/472 (22.9)	134/450 (27.3)	0.81 (0.65–1.00)	0%
CRT vs LC	2/466	79/233 (33.9)	96/233 (41.2)	0.82 (0.65–1.04)	0%
PLR plus SV vs usual care	5/462	58/252 (23.0)	56/210 (26.7)	0.91 (0.67–1.23)	14%
Echocardiography vs usual care	3/214	38/105 (36.2)	46/109 (42.2)	0.72 (0.32–1.61)	70%
IVC vs usual care	3/506	40/253 (15.8)	54/253 (20.9)	0.75 (0.52–1.09)	19%
LC vs usual care*	1/82	15/42 (35.7)	17/40 (42.5)	0.84 (0.49–1.45)	-

*LC* lactate clearance, *ScvO2* central venous oxygen saturation, *CRT* capillary refill time, *PLR* passive leg-raising plus, *SV* stroke volume; *IVC* Inferior vena cava, *echo*: echocardiography

^*^Meta-analysis was not possible

#### Capillary refill time vs lactate clearance

Two RCTs compared CRT with lactate clearance (466 patients). Short-term mortality occurred in 33.9% of 233 patients randomized to capillary refill time and in 41.2% of 233 patients randomized to receive fluid resuscitation driven by lactate clearance, respectively (RR 0.82; 95% CI 0.65–1.04, *I*^2^ 0%) (Fig. 1, panel B and Table 2).

#### Passive leg-raising combined with stroke volume vs usual care

Five RCTs compared the combination of passive leg-raising plus stroke volume with usual care (462 patients); one of these studies was only available in an abstract version [24]. Short-term mortality occurred in 23.0% of 252 patients randomized to passive leg-raising plus stroke volume and in 26.7% of 210 patients randomized to usual care, respectively (RR 0.91; 95% CI 0.67–1.23, *I*^2^ 14%). (Fig. 1, panel C, and Table 2). These results were confirmed after exclusion of the study only available in an abstract version from the analysis (RR 0.84; 95% CI 0.52–1.38, *I*^2^ 34%).

#### Echocardiography vs usual care

Three RCTs compared echocardiography (left ventricular dysfunction, right ventricular dysfunction or tricuspid annular plane systolic excursion, or both and IVC collapsibility) with usual care to guide fluid resuscitation (214 patients). Short-term mortality occurred in 36.2% of 105 patients randomized to echocardiography and in 42.2% of 109 patients randomized to usual care (random effect RR 0.72; 95% CI 0.32–1.61, *I*^2^ 70%) (Fig. 1, panel D, and Table 2).

#### IVC collapsibility vs usual care

Three RCTs compared the IVC collapsibility with usual care (506 patients). Short-term mortality occurred in 15.8% of 253 patients randomized to IVC collapsibility and in 20.9% of 253 patients randomized to usual care to drive fluid resuscitation (RR 0.75; 95% CI 0.52–1.09, *I*^2^ 19%) (Fig. 1, panel E, Table 2).

#### Lactate clearance vs usual care

One RCT compared lactate clearance with usual care to drive fluid resuscitation (82 patients). Short-term mortality occurred in 56.1% of 41 patients randomized to lactate clearance and in 48.8% patients randomized to usual care (RR 0.84; 95% CI 0.49–1.45) (Fig. 1, panel F, and Table 2). A meta-analysis was not possible for this comparison.

### Sub-group analysis

Sub-group analysis was conducted to assess whether the effect of different strategies to guide fluid resuscitation on short-term mortality differed by the clinical setting (ICU vs non-ICU). Consistently with the main analysis, the use of passive leg-raising combined with stroke volume to guide fluid resuscitation was not associated with a significant reduction in short-term mortality compared with usual care, both in ICU (RR 0.81; 95% CI 0.48–1.35, *I*^2^ 0%) and in the non-ICU setting (RR 1.33, 95% CI 0.49–3.61). No effect modification by clinical setting was observed (p int = 0.38), (Figure S3, panel A). The use of lactate clearance-driven fluid resuscitation was not associated with a significant reduction in short-term mortality compared with ScvO₂, both in ICU (RR 0.89, 95% CI 0.67–1.18, *I*^2^ 0%) and in the non-ICU setting (RR 0.82; 95% CI 0.53–1.26). The test for subgroup differences indicated no effect modification by setting (*p* int = 0.74), (Figure S3, panel B). The use of IVC collapsibility was not associated with a reduction in short-term mortality compared to usual care, both in non-ICU setting (RR 0.75; 95% CI 0.01–38.75, I^2^ 35%) (Figure S3, panel C) and in ICU setting (one study, 166 patients; RR 0.65; 95% CI 0.35–1.22). There was no evidence of effect modification by setting (*p* = 0.75), (Figure S3, panel C).

### Sensitivity analysis

Passive leg-raising plus stroke volume was not associated with reduction in short-term mortality regardless of study quality (moderate risk of bias: 1 study; RR 0.50; 95% CI 0.24–1.06; high risk of bias: 4 study; RR 1.03; 95% CI 0.74–1.44, *I*^2^ 0%;), (Figure S4, panel A).

In studies with a low risk of bias, no reduction in short-term mortality was observed with lactate clearance in comparison to ScvO_2_ (2 studies; RR 0.77; 95% CI 0.56–1.05, *I*^2^ 0%), and with IVC collapsibility in comparison to usual care (2 studies; RR 0.82; 95% CI 0.52–1.30, *I*^2^ 52%), (Figure S4, panel B and panel C).

### Length of stay in ICU

LOS in ICU was available for 10 out of the 20 studies, four studies comparing fluid resuscitation driven by lactate clearance vs ScvO2, two comparing passive leg-raising plus stroke volume vs usual care, two comparing CRT vs lactate clearance, and two comparing echocardiography vs usual care. LOS in ICU was shorter when fluid resuscitation was guided by passive leg-raising plus SV compared to usual care (MD: –2.91 days, 95% CI –4.88 to –0.94; *I*^2^ = 0%), (Figure S6, panel B). No difference in LOS in ICU was observed between lactate clearance and ScvO_2_ (MD: –0.20 days, 95% CI −1.97 1.57; *I*^2^ = 77%), between CRT versus lactate clearance (MD: –1.74 days, 95% CI –5.74 to 2.26, *I*^2^ 87%), and between echocardiography versus usual care (MD = –2.84 days, 95% CI –6.13 to 0.46, *I*^2^ = 0%), (Figure S5, panels A, C, and D).

### Network meta-analysis

**Fig. 2:**
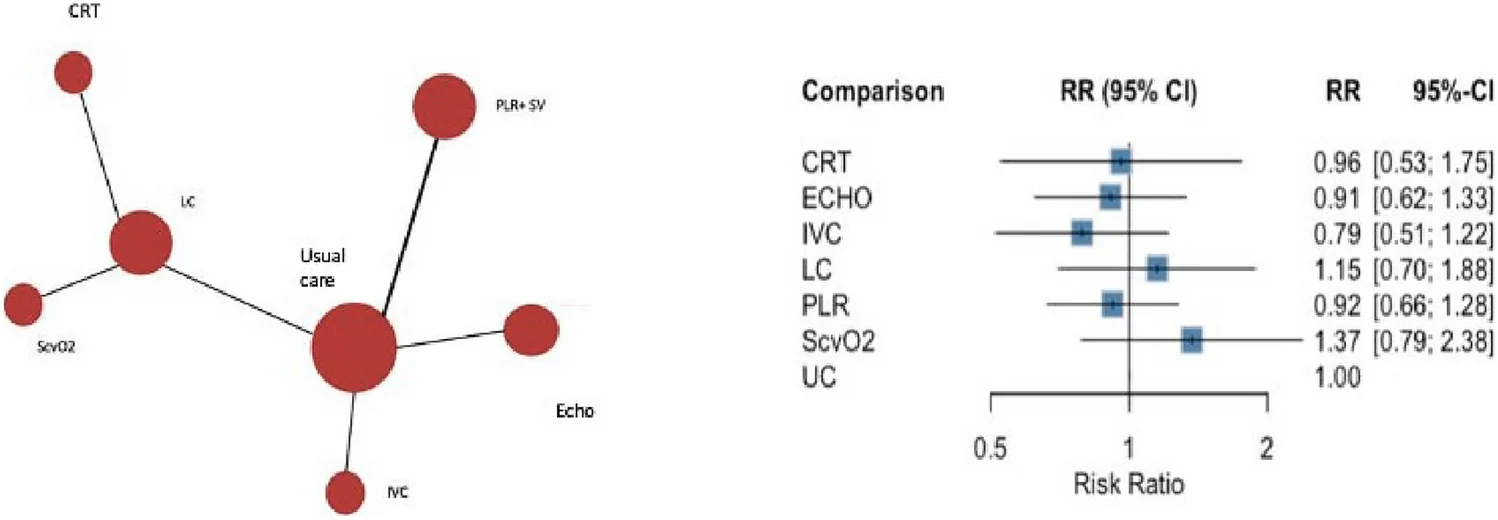
Network meta-analysis: Network graph and main results. Forest plot of network meta-analysis. Risk of short-term death associated with individual strategies for fluid resuscitation compared to usual care

20 selected studies were included in the network meta-analysis, overall assessing the role of seven different interventions to guide fluid resuscitation (lactate clearance in 9 trials, 738 patients; ScvO2 in 6 trials, 922 patients; passive leg-raising plus stroke volume in 5 trials, 252 patients; CRT in 2 trials, 233 patients; usual care in 8 trials, 611 patients; and echocardiography in 3 trials, 106 patients and IVC collapsibility in three trials, 253 patients). Figure 2 shows the geometry of the network plot for short-term mortality.

The main results of the network meta-analysis are presented in Table 3 (random effects model). None of the evaluated strategies for guiding fluid resuscitation was associated with a significant reduction in mortality when compared to usual care, which served as the reference standard treatment (Fig. 2). Network estimates showed a lower mortality with IVC collapsibility compared to CRT (RR 0.83, 95% CI 0.59–0.98), (Table 3).

**Table 3:** Summary of the effect estimates of the interventions to guide fluid resuscitation in short-term mortality

	CRT	ECHO	IVC	LC	PLR + SV	ScvO_2_	Usual care
CRT	-			1.04 (0.52–2.07)	0.58 (0.29–1.16)		
ECHO		-	1.22 (0.58–2.55)	0.99 (0.60–1.64)	0.84 (0.65–1.08)	0.91 (0.62–1.33)	
IVC	*0.83* *(0.59–0.98)*	1.22 (0.58–2.55)	–	0.86 (0.50–1.48)	0.96 (0.53–1.75)		
LC	1.05 (0.52–2.14)	1.15 (0.65–2.05)	0.79 (0.43–1.48)	–	0.91 (0.62–1.33)		
PLR + SV			0.69 (0.36–1.32)	1.25 (0.69–2.26)	–	0.92 (0.66–1.28)	1.37 (0.79–2.38)
ScvO_2_				0.70 (0.46–1.07)	0.79 (0.51–1.22)	–	0.92 (0.66–1.28)
Usual care				0.67 (0.34–1.30)	1.15 (0.70–1.88)	0.67 (0.35–1.28)	–

*LC* lactate clearance; *ScvO2* central venous oxygen saturation; *CRT* capillary refill time; *PLR* passive leg-raising plus; *SV*: stroke volume; *IVC* Inferior vena cava; *echo* Echocardiography; *RR* risk ratio;*CI* confidence interval

Heterogeneity in the network was moderate (*τ*^2^ 0.136). The certainty of the evidence was rated as low, primarily due to the open-loop network geometry which precluded formal assessment of inconsistency in several comparisons and the assessment of incoherence between direct and indirect estimates. The detailed GRADE assessment for each comparison is provided in the (Supplementary Material Table S3).

Ranking analysis suggests that IVC collapsibility ranked the highest, followed by echocardiography, passive leg-raising plus stroke volume, and CRT (Figure S6).

## Discussion

Our meta-analysis including currently available studies on the use of invasive or non-invasive dynamic strategies to guide fluid resuscitation in patients with sepsis or septic shock does not provide evidence of a reduction in short-term mortality for any of these strategies.

These results were confirmed by both standard or network meta-analyses. In addition, the network meta-analysis showed that none of the assessed strategies was associated with reduction in short-term mortality in compared with usual care. ICU-lOS did not differ across invasive or non-invasive dynamic strategies used to drive fluid resuscitation, except for a reduction with passive leg-raising plus stroke volume compared with usual care. However, the evidence on strategies for guiding fluid resuscitation is overall limited and fragmented which difficult to draw definitive conclusions.

In critically ill patients with septic shock, fluid resuscitation together with early antimicrobial treatment is regarded as a key therapeutic measure for initial management as these are associated with reduced mortality [40].

The optimal fluid volume required for appropriate resuscitation is debated, leading to considerable variability in clinical practice, with patients frequently being over-resuscitated.

Fluid overfilling is associated with worse patient outcomes, prolonged need for ventilation, progression to acute kidney injury, and increased mortality [41, 42]. Patients’ hemodynamic response to fluid resuscitation has been measured using static parameters such as central venous pressure or ScvO2 for decades. These measures have increasingly been replaced by the so-called dynamic variables. The 2021 Surviving Sepsis Campaign guidelines for adults with sepsis or septic shock suggest using dynamic parameters to drive fluid resuscitation over static measures alone as these offer real-time insights into patients’ hemodynamic status and allow more accurate assessments of the patient's response to fluid therapy [13].

Based on the results of a recent RCT, the Surviving Sepsis Campaign suggests guiding resuscitation to decrease serum lactate in patients with elevated levels, and using CRT as an adjunct to other measures of perfusion [3, 13, 43]. Both of these non-invasive parameters are particularly suitable for non-ICU setting or in way to intensive care. Other dynamic parameters focusing on the response to passive leg-raising or to fluid bolus administration require experienced ultrasonographers to obtain reliable estimates of stroke volume variation, IVC collapsibility, or other echocardiographic measurements.

Lactate clearance, CRT, and ScvO2 are potential downstream effects of adequate volume resuscitation and serve as indirect assessments of metabolic status, reflecting the concept of "adequate perfusion." Adequate perfusion encompasses the delivery of oxygen to tissues and the ability of tissues to use the delivered oxygen. Stroke volume primarily evaluates one upstream component of tissue perfusion, but it does not account for factors such as hemoglobin concentration or oxygen saturation. Improvement in any upstream or downstream parameter is influenced by volume administration, by antibiotic therapy and infection source control, along with the time required for those interventions to take effect. Our results, showing similar effects in terms of mortality when using lactate clearance or CRT compared with ScvO2, support the suggestions of the Surviving Sepsis Campaign [13]. These findings are clinically relevant as lactate assessment is a simple and reliable test, usually available around the clock; availability of lactate testing can be further improved by the use of validated point-of-care devices [44–47].

CRT is a simple and resource-independent measure but may suffer from significant inter-operator variability [48]. However, when advanced hemodynamic monitoring is not available, CRT may be used to evaluate the effectiveness and safety of volume administration [49, 50].

We identified early mortality as the outcome variable for our study. This does not imply a causal link between measure and survival outcome, but rather between clinical decision informed by the measurement and mortality. For this purpose, rates of adherence to the identified management strategies are essential; therefore, sensitivity analyses by study quality were performed. In addition, mortality may not fully capture nuanced differences in perfusion strategies, which may influence other important clinical aspects, including recovery speed, prevention of organ damage, and the need for invasive support. These outcomes should be addressed in future studies in this setting.

Passive leg-raising in association with stroke volume has been shown to be an accurate measure of fluid responsiveness in the initial assessment of patients with sepsis or septic shock in the ICU and in emergency departments; whether this measure correctly guides fluid management is undefined [51, 52].

In our meta-analysis, passive leg-raising in association with stroke volume was not associated with a reduction in short-term mortality compared with other strategies, but it was associated with reduced ICU-LOS compared with usual care. These results, together with issues concerning the feasibility (time constraints) and reliability (skill level) of stroke volume measurements in emergency situations, question the value of this strategy for guiding fluid resuscitation in sepsis or septic shock.

No clear advantage was observed with the use of ultrasonography to guide fluid resuscitation. Although ultrasound has been proposed as a non-invasive tool to drive fluid resuscitation and its use for monitoring patients with septic shock in intensive care units and in emergency departments has recently become widespread, only limited high-quality data are available [53]. Overall, ultrasonography remains a promising strategy provided optimal parameters are identified and techniques are standardized.

The critical review shows substantial heterogeneity in the assessed studies concerning study design, control strategies, as well as observed mortality rates. 12 of the 20 studies included in our meta-analysis had usual care as the comparator group for guiding fluid resuscitation. Usual care was heterogeneously defined across individual studies, sometimes encompassing invasive monitoring with CVP or pulse pressure variation. The tendency to use invasive monitoring could be due to the high proportion of studies conducted in ICU. However, it also reflects uncertainty about the best strategy to guide patient management in clinical practice. In this context, low heterogeneity observed in most quantitative comparisons is reassuring.

In addition to methodology issues, the use of invasive parameters as CVP has two main drawbacks: first, patient’s exposure to the risk of peri-procedural complications and care to methodological issues, the use of invasive parameters such as CVP has two main drawbacks. First, it exposes patients to the risk of peri-procedural complications and healthcare-associated infections. Second, invasive monitoring requires ICU-level care from the very beginning of fluid resuscitation, which conflicts with everyday clinical practice. [43]

Only three of the included studies were performed outside the ICU setting. Thus, the included patients may not reflect the full spectrum of patients admitted to the emergency department for sepsis or septic shock. However, the initial management of sepsis and septic shock, and in particular fluid replacement, usually begins at the emergency department. In addition, a substantial proportion of patients with sepsis or septic shock is commonly managed outside the ICU. Our results, showing a promising role for non-invasive strategies to guide patient management, may reassure practicing clinicians and healthcare providers. Furthermore, several studies have shown that lactate clearance is associated with decreased mortality in the emergency department [54].

Our study has some limitations. First, some of the included studies had small sample sizes, and none was blinded. Nine of the included studies were categorized as a high risk of bias due to limitations in study design, or incomplete reporting. In addition, five studies were only available in abstract form, thus limiting data verification. These issues reinforce the need for further high-quality studies in this setting. Finally, the limited number of studies conducted outside the ICU may affect the generalizability of the findings to pre-ICU or emergency department populations.

Our study also has some strengths. It assesses all available evidences on the most common non-invasive fluid resuscitation strategies. With the limit of an open loop, we included a network methodology that allowed the comparison of different strategies to identify the optimal fluid management of patients with sepsis and septic shock.

## Conclusion

In conclusion, in the absence of a gold standard, easily available non-invasive dynamic parameters represent promising strategies to guide fluid resuscitation in adult patients with sepsis or septic shock. While these findings need to be supported by further, high-quality evidence, they encourage a potential shift in clinical practice toward the broader use of non-invasive strategies in fluid management protocols. Among non-invasive strategies, lactate clearance, CRT, and assessment of IVC collapsibility are promising, easily obtainable at the bedside and probably worldwide available in this setting.

## Supplementary Information

Below is the link to the electronic supplementary material.

Supplementary file1 (DOCX 4705 KB)

## Data Availability

All data used in this network meta-analysis were extracted from published studies; no new individual-level data were collected. The curated extraction table and the analysis code are provided in the Supplementary Material.
