# Supplementary material for: Comparison of non-invasive strategies to drive fluid resuscitation in sepsis or septic shock: a meta-analysis of RCTs
Source: Intern Emerg Med. 2025 Nov 5;21(7):2637–48. doi: 10.1007/s11739-025-04179-9 (PMC13694371; doi:10.1007/s11739-025-04179-9)
Supplement: Supplementary file 1 [file 11739_2025_4179_MOESM1_ESM.docx]

**Supplementary Material**

**Title**

Comparison of non-invasive strategies to drive fluid resuscitation in sepsis or septic shock.

A meta-analysis of RCTs

**Authors**

Mara Graziani^1^, MD, Laura Gasperini^1^, MD, Claudia Gasperini^1^, MD, Giorgio Maraziti^1^, MD, Gennaro De Pascale ^2^, MD and Cecilia Becattini^1^, MD, PhD.

1. Internal, Vascular and Emergency Medicine – Stroke Unit, University of Perugia, Italy
2. Department of Emergency Science, Anesthesiology, and Resuscitation, Fondazione Policlinico Universitario A. Gemelli IRCCS, Rome, Italy.

**Address for correspondence:**

Mara Graziani, MD

Internal, Vascular and Emergency Medicine – Stroke Unit, University of Perugia, Italy

Phone: 00393339885249

Email: [maragraziani11@gmail.com](mailto:maragraziani11@gmail.com)

List of Contents

Appendix 1. Search strategy

Figure S1. PRISMA 2020 - flow diagram

Figure S2. A) Funnel plot PLR+Sv vs UC; B) funnel-plot LC vs SvcO2; C) funnel plot CRT vs LC: D) funnel plot ECHO vs UC

Figure S3. Subgroups analyses on clinical setting, panel A, panel B and panel C

Figure S4. Sensitivity analyses on study quality , panel A, panel B and panel C

Figure S5. MD ICU- LOS panel A) ScvO_2_ vs UC; panel B PLR + SV vs UC; panel C CRT vs LC; panel D echo vs UC

Figure S6. rank probability histogram

Table S1. Cochrane Collaboration’s tool to assess risk of bias in randomized trials (RoB 2)

Table S2. GRADE Summary of Findings Table – Network Meta-Analysis

Table S3. GRADE framework with CINeMa (Confidence in Network-Meta-analysis) approach.

Appendix 1. Search strategy

Pubmed: ("passive leg rais*" OR "PLR") OR ("lact*") OR ("stroke volume" OR "SV") OR ("ultras*" OR "echo*") OR ("refill" OR "CRT") AND ("sepsis" OR "septic shock")

EMBASE via OVID: **Search 1**: ('passive leg raising'/exp OR 'passive leg raising':ab,ti OR 'passive leg rais*':ab,ti OR PLR:ab,ti)

AND ('sepsis'/exp OR 'septic shock'/exp OR sepsis:ab,ti OR 'septic shock':ab,ti); **Search 2**: ('stroke volume'/exp OR "stroke volume":ti,ab OR "SV":ti,ab)AND('sepsis'/exp OR 'septic shock'/exp OR sepsis:ti,ab OR "septic shock":ti,ab); **Search 3**: ('ultrasonography'/exp OR 'ultrasound'/exp OR 'echocardiography'/exp OR ultrasound*:ti,ab OR echo*:ti,ab OR POCUS:ti,ab) AND ('sepsis'/exp OR 'septic shock'/exp OR sepsis:ti,ab OR "septic shock":ti,ab); **Search 4**: ('capillary refill'/exp OR 'peripheral perfusion'/exp OR "refill":ti,ab OR "capillary refill":ti,ab OR CRT:ti,ab)

AND('sepsis'/exp OR 'septic shock'/exp OR sepsis:ti,ab OR "septic shock":ti,ab)

Trial.gov: (sepsis OR "septic shock") AND ("fluid resuscitation" OR "fluid therapy" OR "fluid management") AND ("length of stay" OR "hospital stay")

Figure S1. PRISMA 2020 - flow diagram

**Identification of studies via databases and registers**

Records removed before screening:

Duplicate records excluded:

(n =2092)

Animal model

(n =4030)

Records identified from:

- Pubmed (n= 14.864 results)
- EMBASE (n=5524)
- ClinicalTrials.gov (n=101)

**Identification**

Records excluded (n=13580)

- Not human: (n=6613)

- not on fluid management: (n=4012)

- Pediatric population: (n=2114)

- Pregnancy population: (n=504)

- Editorial:(n=337)

Records screened

(n =14367)

**Screening**

Records excluded (n=760)

- Not relevant comparator/outcome (n=353)

- Duplicate population (n=208)

- Case series/case report (n=199)

Reports assessed for eligibility

(n =780)

**Included**

Studies included: (n=20)

Figure S2. A) funnel-plot LC vs SvcO2; B) Funnel plot PLR+Sv vs UC; C) funnel plot CRT vs LC: D) funnel plot ECHO vs UC

A)	B)

C)	D)

E)

Figure S3. Subgroups analyses on clinical setting, panel A, panel B and panel C

A. Short-term mortality PLR+ SV vs Usual Care in ICU vs non-ICU setting

B. Short- term mortality LC vs ScvO2 in ICU vs non-ICU setting

C. Short- term mortality IVC vs Usual care in ICU vs non-ICU setting

Figure S4. Sensitivity analyses on study quality

A) Short – term mortality PLR + SV vs Usual Care

B) Short- term mortality LC vs ScvO2

C) Short – term mortality IVC vs usual care

Figure S5. Differences (MD) in LOS in ICU with different strategies for guiding fluid resuscitation

A. LC vs ScvO₂

B. PLR+SV vs UC

C. CRT vs LC

D. Echo vs UC

Figure S6. Rank probability histogram

Table S1. Cochrane Collaboration’s tool to assess risk of bias in randomized trials (ROB 2)

| **Author,year** | **Random sequence generation** | **Allocation concealment** | **Blinding of partecipants and personnel** | **Blinding of outcome assessment** | **Incomplete outcome data** | **Selective reporting** | **Others bias** |
| --- | --- | --- | --- | --- | --- | --- | --- |
| Douglas IS, 2020 | + | - | - | ? | ? | ? | ? |
| Kuan WS,2016 | + | - | - | + | + | + | ? |
| Li G,2019 * | ? | - | - | ? | ? | ? | ? |
| Richard JC,2019 | + | + | - | + | + | + | ? |
| Chen C,2015 | + | ? | - | + | ? | + | ? |
| Jones AE, 2010 | **+** | **+** | **+** | **+** | **+** | **+** | **?** |
| Lyu X, 2015* | **?** | **-** | **-** | **?** | **+** | **?** | **?** |
| Wang T, 2014* | **?** | **-** | **-** | **?** | **?** | **?** | **?** |
| Puskarich MA, 2012 | + | ? | + | + | + | + | ? |
| Tian H, 2012* | ? | - | - | ? | ? | + | ? |
| Yu B, 2013 * | ? | - | - | + | ? | + | ? |
| Hernàndez G,2019 | + | + | + | + | + | + | ? |
| Castro R, 2020 | + | + | + | + | + | + | ? |
| Musikatavorn K,2021 | + | + | + | + | + | + | ? |
| Ghosh S,2024 | ? | ? | - | ? | + | ? | ? |
| Sricharoenchai T, 2024 | + | + | - | + | + | + | + |
| Lanspa MJ, 2018 | + | + | + | ? | + | + | ? |
| Chen H, 2021 | ? | ? | - | + | + | + | ? |
| Li L, 2021 | + | ? | - | ? | + | ? | ? |
| Alhabashy WS, 2021 | + | ? |  | + | + | + | ? |

*Abstract only

Table S2. Results of sensitivity analyses

| Strategies |  | Short term death | |
| --- | --- | --- | --- |
|  | Type of sensitivity | RR 95% CI | I2 |
| PLR+ SV vs UC | ICU | 0.84 (0.60-1.17) | 53% |
|  | non-ICU | 1.33 (0.49-1.23) | -- |
| LC vs ScvO2 | ICU | 0.68(0.53-0.88) | -- |
|  | non-ICU | 0.80(0.52-1.23) | 4% |
| IVC vs UC | ICU | 0.65(0.35-1.22) | -- |
|  | non-ICU | 0.82(0.52-1.30) | 52% |
| PLR+ SV vs UC | Low risk of bias | -- | -- |
|  | Moderate risk of bias | 0.50(0.24-1.06) | -- |
|  | High risk of bias | 1.03(0.74-1.44) | 0% |
| LC vs ScvO2 | Low risk of bias | 0.77 | 0% |
|  | Moderate risk of bias | -- | -- |
|  | High risk of bias | 0.64(0.47-0.87) | 22% |

Table S3. GRADE framework with CINeMa (Confidence in Network-Meta-analysis) approach.

| Comparison | Within-study bias | Across-studies bias | Indirectness | Imprecision | Heterogeneity ° | Incoherence * | Certainty of evidence |
| --- | --- | --- | --- | --- | --- | --- | --- |
| IVC vs Cap refill | Low | Unclear | Minor | High | Moderate | Not Applicable | Low |
| Cap refill vs LC | Moderate | Unclear | Minor | High | Moderate | Not Applicable | Very low |
| ECHO vs IVC | Moderate | Unclear | Minor | Moderate | Moderate | Not Applicable | Low |
| ECHO vs LC | Moderate | Unclear | Minor | High | Moderate | Not Applicable | Very low |
| IVC vs LC | Moderate | Unclear | Minor | High | Moderate | Not Applicable | Very low |
| IVC vs PLR+ SV | High | Unclear | Serious | High | Moderate | Not Applicable | Very low |
| LC vs PLR+ SV | High | Unclear | Minor | High | Moderate | Not Applicable | Very Low |
| LC vs ScvO₂ | High | Unclear | Minor | High | Moderate | Not Applicable | Very Low |
| LC vs Usual care | Moderate | Unclear | Serious | High | Moderate | Not Applicable | Very Low |
| PLR + SV vs ScvO₂ | Moderate | Unclear | Minor | Moderate | Moderate | Not Applicable | Low |
| PLR + SV vs Usual care | High | Unclear | Minor | High | Moderate | Not Applicable | Very Low |
| ScvO₂ vs Usual care | Moderate | Unclear | Serious | High | Moderate | Not Applicable | Very Low |

**The network was open-loop, preventing formal evaluation of inconsistency between direct and indirect estimates.*

*°Heterogeneity was assessed using a globally estimated τ*
